# Author Correction: ΔNp63 regulates a common landscape of enhancer associated genes in non-small cell lung cancer

**DOI:** 10.1038/s41467-022-29349-7

**Published:** 2022-03-25

**Authors:** Marco Napoli, Sarah J. Wu, Bethanie L. Gore, Hussein A. Abbas, Kyubum Lee, Rahul Checker, Shilpa Dhar, Kimal Rajapakshe, Aik Choon Tan, Min Gyu Lee, Cristian Coarfa, Elsa R. Flores

**Affiliations:** 1 Department of Molecular Oncology, H. Lee Moffitt Cancer Center and Research Institute, Tampa, FL USA; 2 Cancer Biology and Evolution Program, H. Lee Moffitt Cancer Center and Research Institute, Tampa, FL USA; 3 Department of Biostatistics and Bioinformatics, H. Lee Moffitt Cancer Center and Research Institute, Tampa, FL USA; 4 Department of Molecular and Cellular Oncology, University of Texas MD Anderson Cancer Center, Houston, TX USA; 5 Department of Molecular and Cell Biology, Baylor College of Medicine, Houston, TX USA

**Keywords:** Cancer stem cells, Non-small-cell lung cancer, Cancer stem cells, Non-small-cell lung cancer

Correction to: *Nature Communications* 10.1038/s41467-022-28202-1, published online 01 February 2022.

In this article, the author name Hussein A. Abbas was incorrectly written as Hussein Abbas. The original article has been corrected.

