# Supplementary material for: ΔNp63 regulates a common landscape of enhancer associated genes in non-small cell lung cancer
Source: Nat Commun. 2022 Feb 1;13:614. doi: 10.1038/s41467-022-28202-1 (PMC8807845; doi:10.1038/s41467-022-28202-1)
Supplement: Supplementary file 1 — Supplementary Information [file 41467_2022_28202_MOESM1_ESM.pdf]

SUPPLEMENTARY INFORMATION

ΔNp63 regulates a common landscape of enhancer associated genes in non-small cell lung cancer

Napoli M., et al.

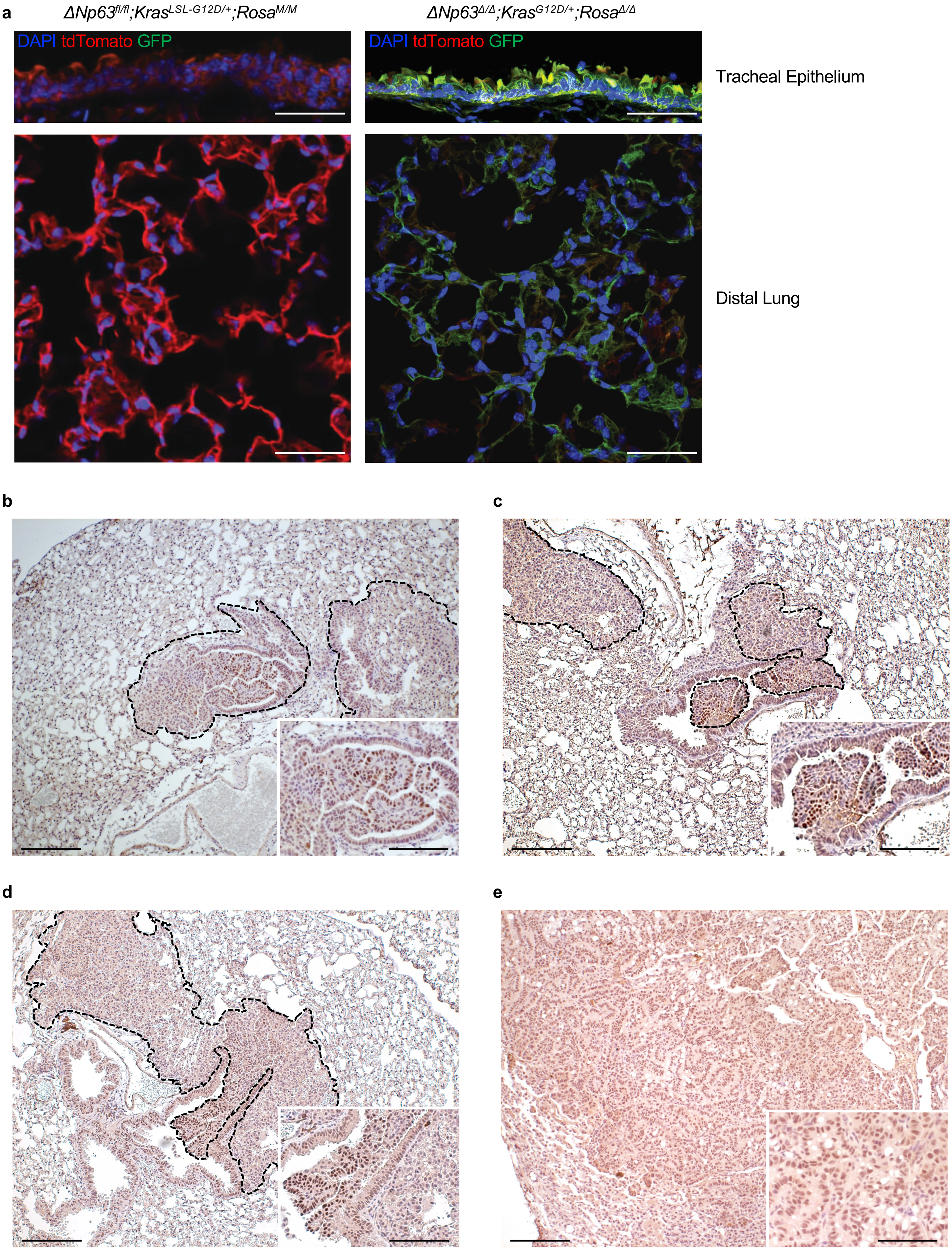
a
$\Delta Np63^{fl/fl};Kras^{LSL\text{-}G12D/+};Rosa^{M/M}$
$\Delta Np63^{\Delta/\Delta};Kras^{G12D/+};Rosa^{\Delta/\Delta}$
DAPI tdTomato GFP
DAPI tdTomato GFP
Tracheal Epithelium
Distal Lung
b
c
d
e

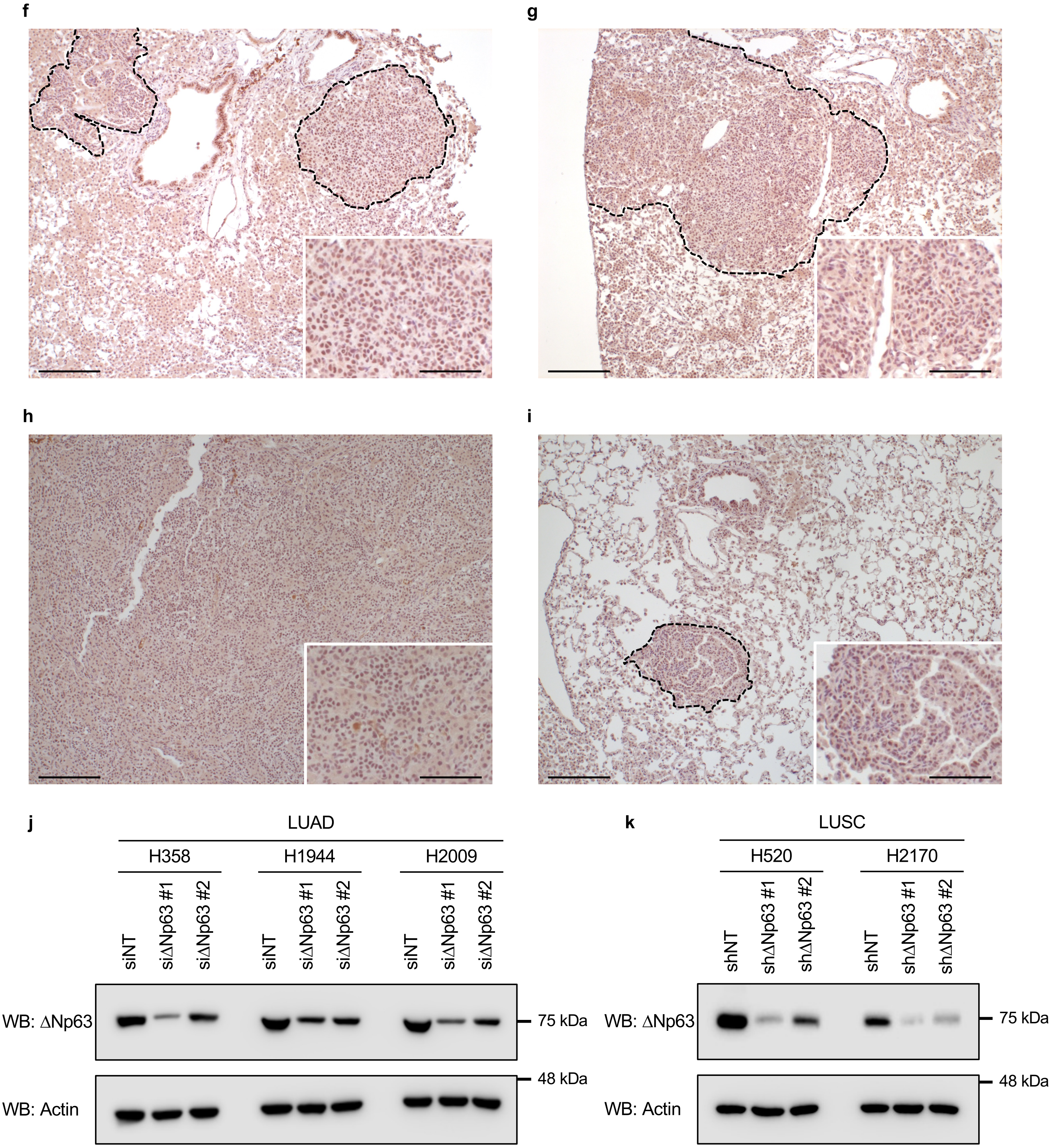

Supplementary Fig 1 ΔNp63 promotes tumor initiation and progression in non-small cell lung cancer. a Representative staining for GFP and tdTomato in tracheal epithelium and distal lungs from *ΔNp63*$^{fl/fl}$;*Kras*$^{LSL\text{-}G12D/+}$;*Rosa*$^{M/M}$ and *ΔNp63*$^{\Delta/\Delta}$;*Kras*$^{G12D/+}$;*Rosa*$^{\Delta/\Delta}$ mice (n = 3). DAPI was used as a counterstaining. Scale bars equal 100 µm. **b,i** Representative images of IHC for ΔNp63 in lung lesions from *Kras*$^{G12D/+}$ mice (n = 3). Scale bars equal 800 µm (bottom left corners) and 200 µm (bottom right corners). Dashed lines outline the areas where the percentage of ΔNp63 positive cells was quantified. **j,k** Representative western blot analysis for the indicated proteins in the indicated LUAD (**j**) and LUSC (**k**) cell lines transfected with the indicated siRNAs (n = 3).

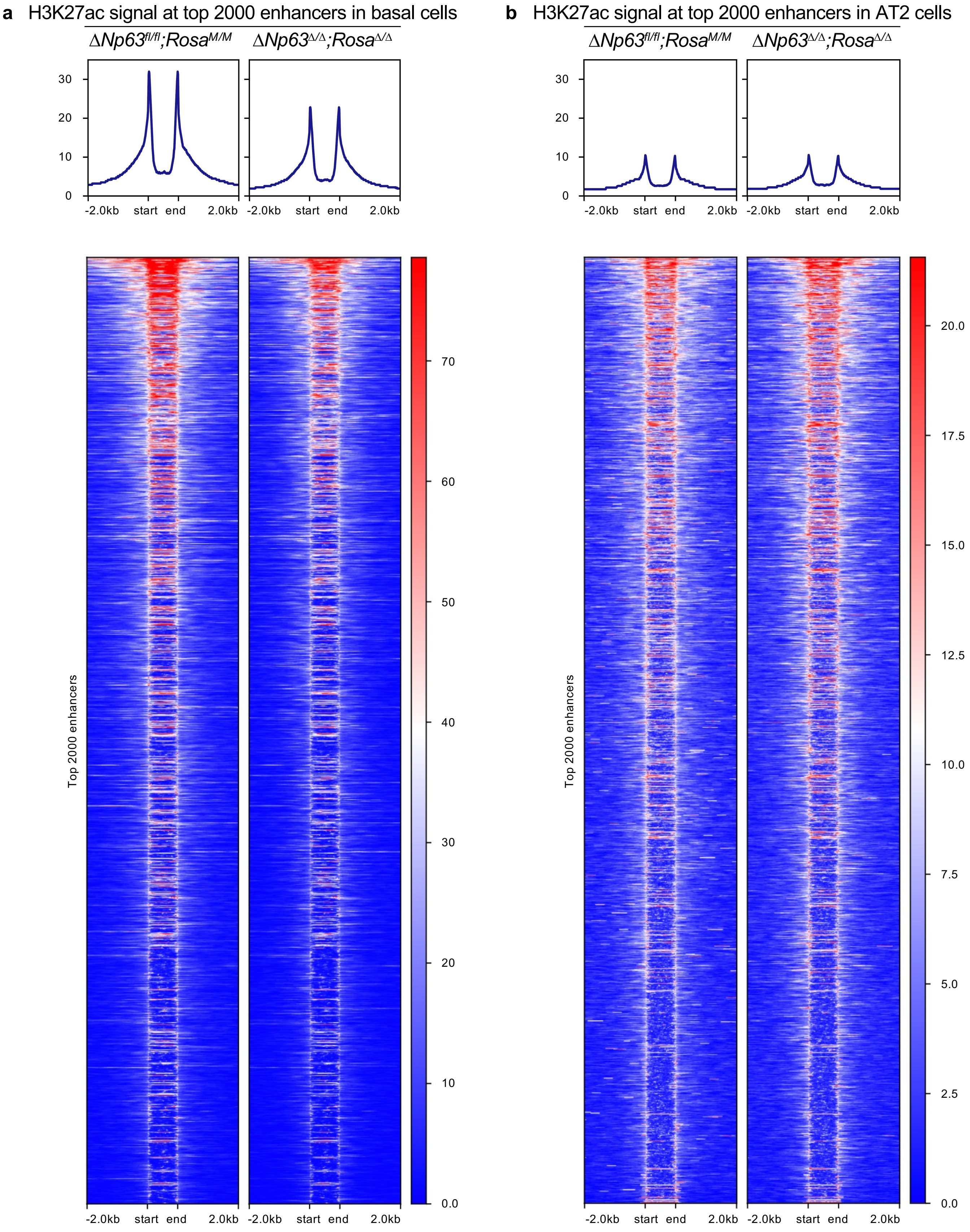

Supplementary Fig 2 ΔNp63 regulates the enhancer landscape of cell identity genes in basal cells and AT2 cells. a,b H3K27ac ChIP-seq signal at the top 2000 enhancer candidates in basal cells (**a**) and AT2 cells (**b**) of the indicated genotypes. Plots and heatmaps are scaled over the enhancer size and included the 2kb before and after the enhancers.

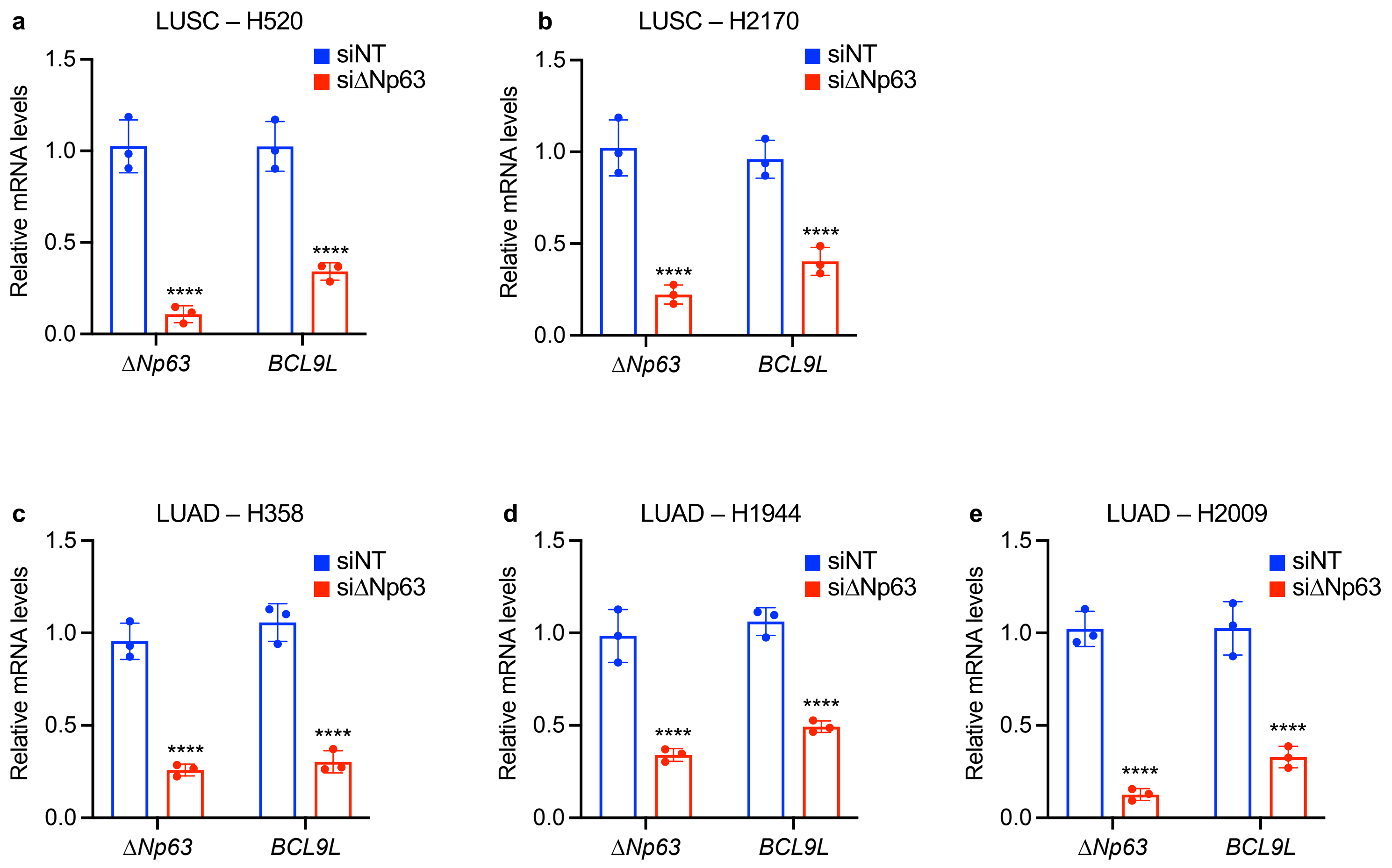

Supplementary Fig 3 ΔNp63 regulates the expression of *BCL9L* in human lung cancer cells. a-e qRT-PCR of *ΔNp63* and *BCL9L* in the indicated human cancer cell lines transfected with the indicated siRNAs. Data are mean ± SD, *n* = 3, **** vs. siNT, *P* < 0.001, two-tailed Student's *t* test.

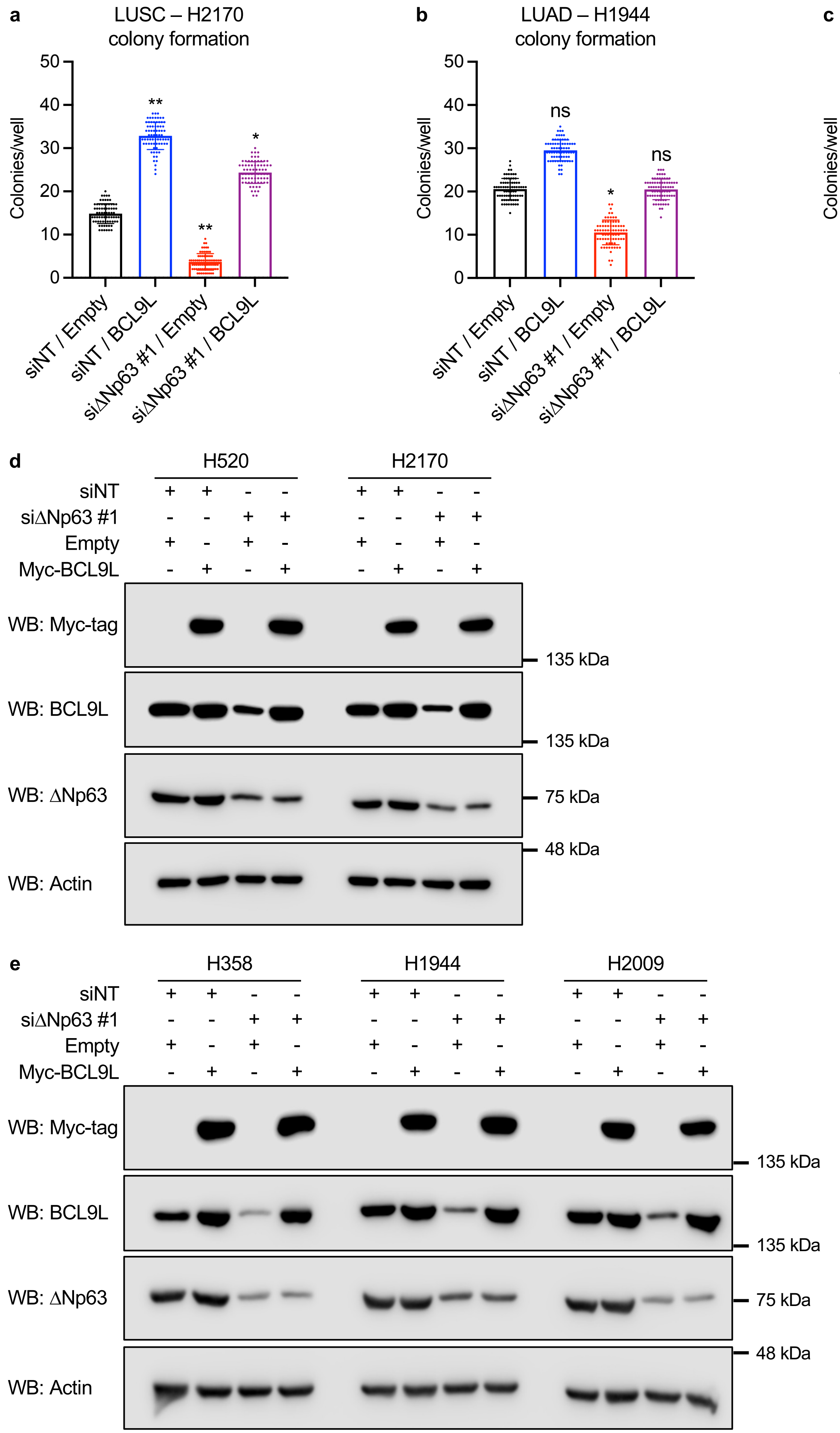

Supplementary Fig 4 *BCL9L* mediates the oncogenic activities of ΔNp63 and is critical for maintenance of LUSC and LUAD. a,c Quantification of colony formation efficiency in soft agar of the indicated cells transfected with the indicated constructs. Boxplots represent the individual data points, median and whiskers (min to max method) are shown, $n = 3$, * = $P < 0.05$, ** = $P < 0.01$, vs. respective siNT / Empty, two-tailed Student's t test. **d,e** Representative western blot analysis for the indicated proteins in the indicated LUSC (**d**) and LUAD (**e**) cell lines transfected with the indicated constructs.
