# Supplementary material for: ΔNp63 regulates a common landscape of enhancer associated genes in non-small cell lung cancer
Source: Nat Commun. 2022 Feb 1;13:614. doi: 10.1038/s41467-022-28202-1 (PMC8807845; doi:10.1038/s41467-022-28202-1)
Supplement: Supplementary file 3 — Description of Additional Supplementary Files [file 41467_2022_28202_MOESM3_ESM.docx]

Description of Additional Supplementary Files

**Title: Supplementary Data 1.**

**Description:** List of differentially enriched pathways in RNA-sequencing data of *ΔNp63^fl/fl^;Rosa^M/M^* vs. *ΔNp63^Δ/Δ^;Rosa^Δ/Δ^* tracheal basal cells. Enriched pathways were determined using the hypergeometric distribution, with significance achieved for FDR-adjusted *P* < 0.05.

**Title: Supplementary Data 2.**

**Description:** List of top 2,000 enhancers in *ΔNp63^fl/fl^;Rosa^M/M^* tracheal basal cells.

**Title: Supplementary Data 3.**

**Description:** Comparison of top 2,000 enhancers in *ΔNp63^fl/fl^;Rosa^M/M^* tracheal basal cells and lung AT2 cells with known enhancers reported on Fantom 5 and Mouse Encode lung datasets.

**Title: Supplementary Data 4.**

**Description:** List of top 2,000 enhancers in *ΔNp63^fl/fl^;Rosa^M/M^* AT2 cells.

**Title: Supplementary Data 5.**

**Description:** Comparison of genes associated to the top 2,000 enhancers in *ΔNp63^fl/fl^;Rosa^M/M^* tracheal basal cells vs. AT2 cells.

**Title: Supplementary Data 6.**

**Description:** Comparison of genes associated to the top 2,000 enhancers in Kras-driven lung adenocarcinomas vs. *ΔNp63^fl/fl^;Rosa^M/M^* AT2 cells.

**Title: Supplementary Data 7.**

**Description:** List of primers utilized for the ChIP assay.

**Title: Supplementary Data 8.**

**Description:** List of qRT-PCR primers.

**Title: Supplementary Data 9.**

**Description:** List of primers utilized for the dCas9 assay.
