# Supplementary material for: ΔNp63 regulates a common landscape of enhancer associated genes in non-small cell lung cancer
Source: Nat Commun. 2022 Feb 1;13:614. doi: 10.1038/s41467-022-28202-1 (PMC8807845; doi:10.1038/s41467-022-28202-1)
Supplement: Supplementary file 13 — Reporting Summary [file 41467_2022_28202_MOESM13_ESM.pdf]

nature portfolio

Corresponding author(s): Elsa R. Flores

Last updated by author(s): Dec 27, 2021

Reporting Summary

Nature Portfolio wishes to improve the reproducibility of the work that we publish. This form provides structure for consistency and transparency in reporting. For further information on Nature Portfolio policies, see our Editorial Policies and the Editorial Policy Checklist.

Statistics

For all statistical analyses, confirm that the following items are present in the figure legend, table legend, main text, or Methods section.

n/a	Confirmed	
☐	☒	The exact sample size (*n*) for each experimental group/condition, given as a discrete number and unit of measurement
☐	☒	A statement on whether measurements were taken from distinct samples or whether the same sample was measured repeatedly
☐	☒	The statistical test(s) used AND whether they are one- or two-sided<br>*Only common tests should be described solely by name; describe more complex techniques in the Methods section.*
☒	☐	A description of all covariates tested
☒	☐	A description of any assumptions or corrections, such as tests of normality and adjustment for multiple comparisons
☐	☒	A full description of the statistical parameters including central tendency (e.g. means) or other basic estimates (e.g. regression coefficient) AND variation (e.g. standard deviation) or associated estimates of uncertainty (e.g. confidence intervals)
☐	☒	For null hypothesis testing, the test statistic (e.g. *F*, *t*, *r*) with confidence intervals, effect sizes, degrees of freedom and *P* value noted<br>*Give P values as exact values whenever suitable.*
☒	☐	For Bayesian analysis, information on the choice of priors and Markov chain Monte Carlo settings
☒	☐	For hierarchical and complex designs, identification of the appropriate level for tests and full reporting of outcomes
☒	☐	Estimates of effect sizes (e.g. Cohen's *d*, Pearson's *r*), indicating how they were calculated

Our web collection on statistics for biologists contains articles on many of the points above.

Software and code

Policy information about availability of computer code

Data collection	Microsoft Excel version 16.16.5
Data analysis	GraphPad Prism version 7.0d, ImageJ

For manuscripts utilizing custom algorithms or software that are central to the research but not yet described in published literature, software must be made available to editors and reviewers. We strongly encourage code deposition in a community repository (e.g. GitHub). See the Nature Portfolio guidelines for submitting code & software for further information.

Data

Policy information about availability of data

All manuscripts must include a data availability statement. This statement should provide the following information, where applicable:
- Accession codes, unique identifiers, or web links for publicly available datasets
- A description of any restrictions on data availability
- For clinical datasets or third party data, please ensure that the statement adheres to our policy

Publicly available data used in this paper were obtained from UCSC (http://genome.ucsc. edu/cgibin/hgGateway) and The Cancer Genome Atlas (TCGA) breast cancer and melanoma datasets (http://www.cancer.gov/about-nci/organization/ccg/research/ structural-genomics/tcga). The RNA-seq data and the ChIP-seq data were deposited to NCBI Gene Expression Omnibus (GEO) repository (series GSE131671, https://www.ncbi.nlm.nih.gov/geo/query/acc.cgi?acc=GSE131671). All the imaging data imaging supporting the findings of this study are available from the corresponding author upon reasonable request. The source data for all the other results are provided as Source Data file with this paper.

Field-specific reporting

Please select the one below that is the best fit for your research. If you are not sure, read the appropriate sections before making your selection.

- [x] Life sciences
- [ ] Behavioural & social sciences
- [ ] Ecological, evolutionary & environmental sciences

For a reference copy of the document with all sections, see nature.com/documents/nr-reporting-summary-flat.pdf

Life sciences study design

All studies must disclose on these points even when the disclosure is negative.

Sample size	For all the in vitro experiments, a minimum of three biological replicates were used per sample. For all the in vivo experiment, a minimum of five mice were used per each group. No statistical method was used to determine the sample/group size.
Data exclusions	No technical replicates were excluded from any presented data.
Replication	Experiments were successfully performed at least three times. All replication attempts were successful.
Randomization	Mice were randomly allocated into different experimental groups before being injected with the cell lines of interest. For experiments involving cellular and biological studies, three independent experiments have been performed, allocating randomly cells in experimental groups.
Blinding	Tumor volume assessment was blinded. The IHC signals and the proportion of positive tissue were measured blindly. For most of the other experiments, the results were quantified and appropriate statistical tests were performed to evaluate difference and statistical significance.

Reporting for specific materials, systems and methods

We require information from authors about some types of materials, experimental systems and methods used in many studies. Here, indicate whether each material, system or method listed is relevant to your study. If you are not sure if a list item applies to your research, read the appropriate section before selecting a response.

Materials & experimental systems

n/a	Involved in the study	
[ ]	[x]	Antibodies
[ ]	[x]	Eukaryotic cell lines
[x]	[ ]	Palaeontology and archaeology
[ ]	[x]	Animals and other organisms
[x]	[ ]	Human research participants
[x]	[ ]	Clinical data
[x]	[ ]	Dual use research of concern

Methods

n/a	Involved in the study	
[ ]	[x]	ChIP-seq
[x]	[ ]	Flow cytometry
[x]	[ ]	MRI-based neuroimaging

Antibodies

Antibodies used

The following primary antibodies were utilized for IF and/or IHC: ΔNp63 (ab172731, Abcam), GFP (ab13970, Abcam), Ki67 (ab15580, Abcam), cytokeratin 5 (ab53121, Abcam), acetylated tubulin (T7451, Sigma-Aldrich), mucin 5ac (MA1-21907, ThermoFisher Scientific), cleaved caspase 3 (9661, Cell Signaling), CC10 (sc-25555, Santa Cruz), SPC (sc-7705, Santa Cruz), and NGFR (ab8874, Abcam).

The following primary antibodies were used for western blot analysis: ΔNp63 (619002, BioLegend), BCL9L (PA5-61946, ThermoFisher), Myc-tag (2276, Cell Signaling), and Actin (4967S, Cell Signaling).

For the low cell number ChIP assays, the following antibodies were used: H3K27ac (39193, Active Motif), RNA polymerase 2 (39097, Active Motif), and ΔNp63 (sc-8609, Santa Cruz).

BASCs and AT2 cells were isolated using the following antibodies: CD31-APC (551262, BD), CD45-APC (559864, BD), EpCAM-PE-Cy7 (118216, BioLegend), SCA-1-APC-Cy7 (560654, BD).

Validation

Antibody: Actin (4967S, Cell Signaling)
Species reactivity: multiple species including human and mouse
Application validated by manufacturer: WB

Antibody: acetylated tubulin (T7451, Sigma-Aldrich)
Species reactivity: multiple species including human and mouse

Applications validated by manufacturer: IHC, IF, WB

Antibody: BCL9L (PA5-61946, ThermoFisher)
Species reactivity: human
Applications validated by manufacturer: IHC, IF, WB

Antibody: CC10 (sc-25555, Santa Cruz)
Species reactivity: mouse
Applications validated by manufacturer: IF, IP, WB

Antibody: CD31-APC (551262, BD)
Species reactivity: mouse
Application validated by manufacturer: FACS

Antibody: CD45-APC (559864, BD)
Species reactivity: mouse
Application validated by manufacturer: FACS

Antibody: cleaved caspase 3 (9661, Cell Signaling)
Species reactivity: multiple species including human and mouse
Applications validated by manufacturer: IHC, IF, WB

Antibody: cytokeratin 5 (ab53121, Abcam)
Species reactivity: multiple species including human and mouse
Applications validated by manufacturer: IHC, IF, WB

Antibody: ΔNp63 (ab172731, Abcam)
Species reactivity: human and mouse
Application validated by manufacturer: IHC

Antibody: ΔNp63 (619002, BioLegend)
Species reactivity: human
Application validated by manufacturer: WB

Antibody: ΔNp63 (sc-8609, Santa Cruz)
Species reactivity: multiple species including human and mouse
Applications validated by manufacturer: IF and WB

Antibody: EpCAM-PE-Cy7 (118216, BioLegend)
Species reactivity: mouse
Application validated by manufacturer: FACS

Antibody: GFP (ab13970, Abcam)
Species reactivity: all
Applications validated by manufacturer: IHC, IF, WB

Antibody: H3K27ac (39193, Active Motif)
Species reactivity: mouse
Application validated by manufacturer: ChIP

Antibody: Ki67 (ab15580, Abcam)
Species reactivity: human and mouse
Applications validated by manufacturer: IHC and IF

Antibody: mucin 5ac (MA1-21907, ThermoFisher Scientific)
Species reactivity: multiple species including human and mouse
Application validated by manufacturer: IHC

Antibody: Myc-tag (2276, Cell Signaling)
Species reactivity: all
Applications validated by manufacturer: IHC, IF, WB

Antibody: NGFR (ab8874, Abcam)
Species reactivity: human and rat
Application validated by manufacturer: IHC

Antibody: RNA polymerase 2 (39097, Active Motif)
Species reactivity: human and mouse

Application validated by manufacturer: ChIP

Antibody: SCA-1-APC-Cy7 (560654, BD)
Species reactivity: mouse
Application validated by manufacturer: FACS

Antibody: SPC (sc-7705, Santa Cruz)
Species reactivity: multiple species including human and mouse
Application validated by manufacturer: IF

Eukaryotic cell lines

Policy information about cell lines

Cell line source(s)	The KrasG12D/+ expressing human lung cancer cell lines (H520, H2170, H358, H1944, and H2009) and the 293T cell line were obtained from ATCC, while the primary tracheal basal cells and AT2 cells were isolated from the indicated GEMM mice.
Authentication	All the cell lines were authenticated by STR profiling by the MD Anderson Cell Line Authentication Service.
Mycoplasma contamination	All the cell lines used were mycoplasma negative.
Commonly misidentified lines (See ICLAC register)	No commonly misidentified cell lines were used.

Animals and other organisms

Policy information about studies involving animals; ARRIVE guidelines recommended for reporting animal research

Laboratory animals	All the GEMM mice used in this study were in a C57BL/6 background. All the mice used in this study for the xenograft experiments were athymic nu/nu mice. Mice were aged until 6-8 weeks old before being utilized in experiments. Both male and female mice were used at equal ratios. Mice were housed pathogen-free and ventilated cages, and allowed free access to irradiated food and autoclaved water ad libitum in a 12h light/dark cycle, with room temperature at 21±2°C and humidity between 45 and 65%.
Wild animals	The study did not involve wild animals.
Field-collected samples	The study did not involve field-collected samples.
Ethics oversight	All procedures were approved by the IACUC at the MD Anderson Cancer Center and the H. Lee Moffitt Cancer Center & Research Institute.

Note that full information on the approval of the study protocol must also be provided in the manuscript.

ChIP-seq

Data deposition

☒ Confirm that both raw and final processed data have been deposited in a public database such as GEO.

☒ Confirm that you have deposited or provided access to graph files (e.g. BED files) for the called peaks.

Data access links<br>*May remain private before publication.*	The ChIP-seq data were deposited to NCBI Gene Expression Omnibus (GEO) repository (series GSE131671).
Files in database submission	GSM3813489<br>GSM3813490<br>GSM3813491<br>GSM3813492<br>GSM3813493<br>GSM3813494<br>GSM3813495<br>GSM3813496<br>GSM5692007<br>GSM5692008<br>GSM5692009<br>GSM5692010<br>GSM5692011<br>GSM5692012<br>GSM5692013<br>GSM5692014<br>GSM5692015<br>GSM5692016<br>GSM5692017

GSM5692018
GSM5692019
GSM5692020

Genome browser session (e.g. UCSC): no longer applicable

Methodology

Replicates: The ChIP-seq was performed by pooling either primary basal or AT2 cells collected from at least 5 ΔNp63fl/fl;RosaM/M mice,

Sequencing depth: At least 30 millions reads (75 nt, single end) per samples were sequenced.

Antibodies: H3K27ac (39193, Active Motif), RNA polymerase 2 (39097, Active Motif), and ΔNp63 (sc-8609, Santa Cruz).

Peak calling parameters: Sequencing was analysed with MACS to call peaks with q < 0.05 and mapped using bowtie2 against the mouse genome build UCSC mm10.

Data quality: The data quality was assessed using FastQC.

Software: ChIP-Seq signal maps were generated using BEDTools and visualized using the Integrative Genome Viewer .
